# Does body mass index distinguish motor proficiency, social and emotional maturity among adolescent girls?

**DOI:** 10.1186/s12887-023-04443-x

**Published:** 2023-12-06

**Authors:** Georgian Badicu, Seyed Hojjat Zamani Sani, Zahra Fathirezaie, Mohaddese Esmaeili, Júlio Cesar Bassan, Francisco Tomás González-Fernández, Fatma Hilal Yagin, Abdullah F. Alghannam, Stefania Cataldi, Francesco Fischetti, Gianpiero Greco

**Affiliations:** 1 Department of Physical Education and Special Motricity, Faculty of Physical Education and Mountain Sports, Transilvania University of Braşov, Braşov, 500068 Romania; 2 Motor Behavior Department, Physical Education and Sport Sciences Faculty, University of Tabriz, Tabriz, 51666 Iran; 3 Postgraduate Program in Physical Education, Universidade Tecnológica Federal do Paraná, Curitiba, Brazil; 4 Department of Physical Education and Sport, Faculty of Education and Sport Sciences, Campus of Melilla, University of Granada, Melilla, 52006 Spain; 5 Department of Biostatistics and Medical Informatics, Faculty of Medicine, Inonu University, Malatya, 44280 Turkey; 6 Lifestyle and Health Research Center, Health Sciences Research Center, Princess Nourah bint Abdulrahman University, Riyadh, Saudi Arabia; 7 Department of Transitional Biomedicine and Neuroscience (DiBraiN), University of Study of Bari, Bari, 70124 Italy

**Keywords:** Overweight, Underweight, Motor competence, Social competence, Emotional competence

## Abstract

**Background:**

The objective of this study was to investigate whether different body mass index (BMI) groups could serve as a distinguishing factor for assessing motor proficiency and social and emotional maturity in adolescent girls.

**Methods:**

140 girls ranging from 12 to 14.5 years old were selected from the schools of Tabriz city, Iran. After their height and weight were measured to calculate body mass index, they completed the following questionnaires: Bruininks-Oseretsky Test of motor proficiency, Second Edition,Vineland Social Maturity Scale, and Emotional Maturity scale.

**Results:**

normal-weight girls had a meaningful advantage against overweight and underweight participants in the gross motor factor of motor proficiency (*p* = 0.004), but there wasn’t a meaningful difference in the fine motor *p* = 0.196) and coordination factors (*p* = 0.417). Also, social maturity showed an advantage of normal and underweight adolescent girls in the self-help dressing factor (*p* = 0.018), while the locomotion skills (*p* = 0.010) factor revealed a better performance of normal weight and overweight groups over underweight adolescents. No significant differences were observed in the emotional maturity subscales (*p* = 0.63) between the groups.

**Conclusions:**

The present study demonstrates that BMI has a direct influence on adolescents’ gross motor proficiency and social maturity.

## Introduction

### Body mass index

Overweight incidence has widely risen worldwide among adolescents [1]. In 1980, 5% of adolescents were obese, but in recent years, it has risen to 21% [2]. With these interpretations and the importance of increasing body fat in girls during adolescence in order to maintain regular menstrual cycles [3], it seems important to investigate the side effects and delayed influences of this weight gain, especially in adolescent girls. Despite the lack of identification and differentiation of different types of body tissues in the formation of body weight, body mass index (BMI) is well-established and used to characterize being overweight and obese in adolescents [4]. Also, the report of Minnesota University showed that the shape index W/Ht2 has the highest relationship with fatness among the other factors such as the skinfolds and fat percentage by underwater weighing [5].

### Aspects of development

#### Motor development

Development encompasses various aspects, and one of the most crucial among them is motor development., which is affected by obesity [6]. According to the definitions, changes in motor behavior during life and the processes underlying these changes is called “motor development”. The three main stages of motor development are infancy, childhood, and adolescence and body weight is an important target for understanding developmental outcomes in youths [7]. Movements that are mostly controlled by a muscle or a group of large muscles (such as walking and running) are called gross movements and movements that are mostly controlled by small muscles (drawing) are called fine movements [8]. Several kinds of research have been conducted to measure the correlation between motor development and obesity or BMI. Amouian et al. [9] illustrated that the motor development in the obese and overweight group was at a low level compared to other groups and there was a considerable difference between the obese and overweight group and the normal group and the underweight and overweight, obese and normal groups were dramatically different. In parallel, it is shown that pre-obese and obese children have certain problems in motor dimension in the segment which is related to the showing of muscle strength and power, speed and balance [10].

Also, Lopez et al. [11] concluded that normal-weight children showed higher motor coordination scores than overweight children among both genders. Also, obese children had the lowest motor coordination scores among all three groups. Furthermore, Logan et al. [12], showed that overweight and obese children performed worse in performing fundamental movement skills. They stated that there is a direct relationship between body mass index and children’s fundamental movement skills. So, the first aim of the study was to investigate the differences in motor proficiency of different BMI groups (underweight, normal, and overweight) among adolescent girls.

#### Social development

Another aspect of development is social development. During the developmental process, as youth move from childhood into adolescence, they need to get socialization messages in different ways [13]. Socialization is a dual process of development, through which people learn who they are and how to communicate with the society around them in which they live [8]. In other words, it is a process by which individuals acquire the knowledge, skills, attitudes, and behaviors to assume a role [14]. Movement activities that people choose affect people’s ability to acquire social competence [15]. In a study by Letsari et al. [16] in Obese children in Surakatra, Indonesia, they showed that social immaturity in the group of obese children was 2 to 4 times more than that of normal-weight children. In a cross-sectional study by Whittingham et al. [17] on 122 children, they cited that there is a correlation between motor abilities and social maturity of these children. Also, research conducted by Verdejo-García et al. [18] showed that being overweight reduces the activation of the brain area that is necessary for social decision-making, and in this connection, the fear between social communication and social maturity is reduced. So, the second aim of the study was the investigate the differences of social development in different BMI groups (underweight, normal and overweight) among adolescent girls.

#### Emotional development

Social development is a flow during which a person continuously strives to create the ability to resist delays in satisfying needs, and during which a person can tolerate a number of emotions with different levels [19]. Madrona et al. [20] investigated the effect of body mass on motor, cognitive, and social-emotional skills. There were significant results among the group of girls in all the studied skills, especially between the group of overweight and obese girls and the group with normal weight. However, this difference was related to other factors, between the groups of boys. So, the last aim of the present research was the investigate the differences in Emotional maturity in different BMI groups (underweight, normal, and overweight) among adolescent girls.

Based on the research background and stated aims, the main hypothesis of the study is that there is a correlation between body mass index and the development of motor, social, and emotional skills in adolescents. It is likely that varying levels of body mass index can differentiate the aforementioned abilities in adolescent development.

## Materials and methods

### Subjects

The present research was a causal-comparative study. The statistical population of this study was 140 adolescent girls aged 12 to 14.5 who were selected among the teenagers by cluster random sampling from four schools in District 1, Tabriz City.

G*Power software version 3.1.9.4 was used to estimate the sample size. The maximum number of predictors in our study among three MANOVA tests was used. Additionally, the effect size was estimated to be 0.2 based on the literature review, the alpha error probability was 0.05, the number of groups was three, and the power of the test was 0.95. Therefore, the total sample size was calculated as 122.

### Procedures

After coordination with the departments of education, school management, and sports clubs, a suitable environment was provided for research and data collection. In the beginning, the weight and height of the students were measured to calculate the subjects’ BMI and determine the groups: The obese group had a BMI of more than 25, n = 50; the normal group, BMI = 18.5–24.9, n = 50, and the underweight group had a BMI below 18.5, n = 40). Measurement of height and weight continued until reaching the number of people in each group.The criteria for entering the research included the following, which were collected by self-report:

Being 12 to 14.5 years old, the absence of motor and mental problems, long-term insomnia, and illness, not being a professional athlete and lack of regular physical activity.

### Instruments

#### Bruininks-Oseretsky Test of motor proficiency, second edition (BOT-2)

This test measures motor proficiency in four composite factors based on hand predominancy (‘Fine Manual Control’ and ‘Manual Coordination’) or whole/gross body functions (‘Body Coordination’ and ‘Strength and Agility’). In the full version, 53 items were arranged into eight subscales for the motor proficiency assessment of children and adolescents [21]. Dietz et al. [22] found the validity of this test to be 0.86 and its reliability coefficient was 0.90. The sensitivity = 0.91 and specificity = 0.93 of this tool have been confirmed [23].

#### Vineland social maturity scale

This test was developed by Doll in 1936. This is a standardized tool for the assessment of social competence from childhood to adulthood as a developmental process that leads to the social abilities of persons [24]. This scale has 117 items which include eight subscales: self-help eating (12 items), self-direction (12 items), self-help dressing (15 items), locomotion skills (10 items), general self-help ability (14 items), occupation (22 items), socialization (17 items), and communication skills (15 items) [25]. Also, the validity and reliability of that scale is confirmed in recent years [26]. The sensitivity = 82% and specificity = 81% of this tool in determining mental age has been confirmed [27].

#### Emotional maturity scale (EMS)

The emotional maturity scale in 48 items, is a self-reporting five-point scale that measures different dimensions of emotional maturity such as emotional progression, emotional stability, personality integration, social adjustment, and independence. The scale test-retest reliability and validity were 0.75 and 0.64 respectively [28].

The sensitivity of this tool is defined conceptually. It is shown that adolescents with high emotional maturity showing significantly high stress may be attributed to the fact that naturally the emotionally matured people are highly sensitive and more concerned to the happenings of the world and get themselves involved in each and every aspect of life which in turn makes them to feel more stressful [29].

#### BMI

In assessing obesity and overweight in children, weight and height scales are often combined and the “body mass index” scale is obtained. Body mass index was calculated by weight (kg) / [height (m)]^2^. Junior et al. [30] cited that estimates of prevalence of obesity among adolescents using different BMI classification criteria were similar and highly specific (83.6–98.8%) for both sexes, and very sensitive for males (85.4–91.7%).

### Statistical analysis

Three multivariate analysis of variance (MANOVA) was used to show the probable differences in motor proficiency, social and emotional maturity among three BMI groups with IBM® SPSS® Statistics software version 23. For each variable, A MANOVA was used. First, description statistics were shown in a separate table (Tables 1 and 2, and 3). Then MANOVA statistics were used to identify the differences in study variables among BMI groups (Tables 4 and 5). In addition, F statistics, significant level, and Partial Eta Squared were assessed. Partial eta-square were used to show the magnitude effect size (trivial < 0.2, small ≥ 0.2; medium ≥ 0.5; large ≥ 0.8 and above). In the variables that were shown to be significant, follow-up statistics including pairwise comparisons (Tables 6 and 7) were performed (LSD post-hoc tests).

**Table 1:** Descriptive statistics of motor proficiency factors among groups

Variables	Group	Mean	SD	Kolmogorov- Smirnov		
				statistic		P
Gross motor	Overweight	31.84	4.20	0.089		0.212
	Normal	34.28	3.94	0.104		0.209
	Underweight	32.18	3.31	0.102		0.209
Fine motor	Overweight	28.14	5.11	0.095		0.210
	Normal	28.10	5.75	0.034		0.420
	Underweight	26.33	4.86	0.042		0.254
Coordination	Overweight	4.88	0.68	0.078		0.221
	Normal	5.10	0.78	0.120		0.140
	Underweight	4.97	1.02	0.101		0.210

SD: standard deviation

**Table 2:** Results of the MANOVA test in the motor proficiency variables

Source	Dependent Variable	Type III Sum of Squares	df	Mean Square	F-value	*p*-value	Partial Eta Squared
Group	Gross motor	171.21	2	85.60	5.70	0.004	0.07
	Fine motor	92.09	2	46.04	1.64	0.196	0.02
	Coordination	1.21	2	0.60	0.87	0.417	0.01

**Table 3:** Pairwise Comparisons of the BMI groups in the gross motor skills

Dependent Variable	(I) Group	(J) Group	Mean Difference (I-J)	SE	*p*-value
Gross motor	Overweight	Normal	-2.44^*^	0.77	0.002
	Overweight	Underweight	-0.33	0.82	0.684
	Normal	Underweight	2.10^*^	0.82	0.011

SE: standard error

**Table 4:** Descriptive statistics of social maturity factors among groups

Variables	Group	Mean	SD	Kolmogorov- Smirnov	
				statistic	P
Self-direction	Overweight	10.06	2.20	0.971	0.252
	Normal	10.14	1.68	0.717	0.430
	Underweight	10.22	2.36	0.845	0.341
Occupation skills	Overweight	18.32	1.78	0.960	0.220
	Normal	18.27	1.37	0.912	0.270
	Underweight	17.82	1.93	0.909	0.270
Socialization skills	Overweight	15.37	1.62	0.943	0.211
	Normal	15.61	1.44	0.789	0.367
	Underweight	15.23	1.22	0.961	0.250
Self-help dressing	Overweight	12.88	0.32	0.881	0.342
	Normal	12.97	0.11	0.894	0.356
	Underweight	13.00	0.00	0.714	0.382
Self-help eating	Overweight	11.99	0.07	0.879	0.334
	Normal	11.93	0.24	0.945	0.260
	Underweight	11.98	0.07	0.779	0.350
Locomotion skills	Overweight	8.26	0.60	0.886	0.252
	Normal	8.24	0.73	0.770	0.345
	Underweight	7.87	0.58	812	0.270
Communication skills	Overweight	10.36	1.41	0.944	0.221
	Normal	9.83	1.16	0.632	0.470
	Underweight	9.92	1.44	0.718	0.375
General Self-help	Overweight	15.00	0.00	0.870	0.330
	Normal	15.00	0.00	0.946	0.215
	Underweight	15.00	0.00	0.900	0.280

SD: standard deviation

**Table 5:** Results of the MANOVA test in the social maturity variables

Source	Dependent Variable	Type III Sum of Squares	df	Mean Square	F-value	*p*-value	Partial Eta Squared
Group	Self-direction	0.60	2	0.30	0.07	0.933	0.001
	Occupation skills	6.37	2	3.18	1.10	0.334	0.016
	Socialization skills	3.26	2	1.63	0.77	0.464	0.011
	Self-help dressing	0.36	2	0.18	4.15	0.018	0.057
	Self-help eating	0.11	2	0.05	2.18	0.116	0.031
	Locomotion skills	4.02	2	2.01	4.78	0.010	0.065
	Communication skills	7.84	2	3.92	2.18	0.116	0.031
	General Self-help	0.001	2	0.001	0.001	0.999	0.0001

**Table 6:** Pairwise Comparisons

Dependent Variable	(I) Group	(J) Group	Mean Difference (I-J)	SE	*p*-value
Self-help dressing	Overweight	Normal	-0.09	0.042	0.033^*^
		Underweight	-0.12	0.044	0.008^*^
	Normal	Underweight	-0.03	0.044	0.500
Locomotion skills	Overweight	Normal	0.02	0.130	0.878
		Underweight	0.38	0.138	0.006^*^
	Normal	Underweight	0.36	0.138	0.009^*^

SE: standard error

**Table 7:** Descriptive statistics of emotional maturity factors among groups

Variables	Group	Mean	SD	Kolmogorov- Smirnov	
				statistic	statistic
Emotional stability	Overweight	20.1800	7.46609	0.984	0.721
	Normal	19.1000	5.99404	0.920	0.744
	Underweight	19.9500	6.04661	0.812	0.854
Emotional progression	Normal	18.1200	5.68794	0.961	0.715
	Underweight	17.2800	5.66421	0.861	0.880
	Overweight	17.6500	5.26990	0.816	0.850
Social adjustment	Underweight	19.6000	6.27922	0.925	0.750
	Overweight	20.1400	4.77241	0.980	0.720
	Normal	19.1750	4.47149	0.859	0.847
Personality integration	Overweight	15.7600	6.14970	0.970	0.743
	Normal	15.3000	4.53220	0.946	0.724
	Underweight	16.9750	4.29363	0.889	0.792
Independence	Normal	17.6200	4.53058	0.866	0.850
	Underweight	17.5600	3.62621	0.921	0.751
	Overweight	17.2000	3.17199	0.790	0.687

SD: standard deviation

## Results

As can be seen, the research results are presented in three separate sections for motor proficiency, social and emotional maturity.

### Motor proficiency

Multivariate tests of motor proficiency showed a significant difference between groups (Wilks’ Lambda value = 0 0.875, F = 3.11, *p* = 0 0.006). The following results showed that only gross motor skills have significant differences among BMI groups (Table 4).

In the following, pairwise comparisons of the groups showed normal BMI group had a higher score for gross motor skills than others (Table 6).

The first hypothesis did not show significant differences in fine motor and coordination factors among different groups of BMI. However, gross motor revealed a meaningful advantage of normal weight against overweight and underweight adolescent girls. It should be noted that underweight and overweight adolescents did not have a significant difference in sum between them.

### Social maturity

In addition, a multivariate test of social maturity showed a significant difference between groups (Wilks’ Lambda value = 0.76, F = 2.75, *p* = 0 0.001). The following results showed that only gross motor skills have significant differences among BMI groups (Table 5).

Pairwise comparisons of the groups showed overweight BMI group had a lower score for Self-help dressing than others. Also, the underweight BMI group had a higher score in locomotion skills than other groups (Table 7).

The second hypothesis of social maturity had two main results. First, both the normal and underweight groups had better outcomes than the overweight in self-help dressing. The second item was the dominance of both normal and overweight groups over underweight adolescents in locomotion skills. Other items of the scale did not show significant results.

### Emotional maturity

Last but not least, a multivariate test of emotional maturity showed no significant difference between groups (Wilks’ Lambda value = 0 0.94, F = 0 0.79, *p* = 0.63). So, the third hypothesis did not show a meaningful difference in emotional maturity among groups.

## Discussion

This study pointed out that BMI can discern how each factor (motor proficiency, social and emotional maturity) is affected by overweight, normal, or under-weight in adolescent girls, and which one is the biggest determinant.

The results of the first hypothesis in the gross motor factor confirmed that motor development is affected by obesity, and this finding is consistent with Hamilton et al. [6], where overweight children were delayed in performance of gross and fine motor skill. Our study focused on adolescent girls, while the mentioned study had male and female children as participants. Boys had better results in gross motor activities, but girls performed better in fine motor skills. Notwithstanding, overweight adolescent girls did not perform well in gross motor skills in our study. It should be noted that the present study did not provide a significant difference in fine motor skills between adolescent girls with varying BMI.

Our study is also in line with Amouian et al. [9] where obese and overweight children were at a lower level of gross motor development compared to other groups. The mentioned study found a significant and inverse correlation between BMI and locomotor skills such as jumping, running, and dancing (gross motor skills). Well-developed skills can help children and adolescents to move more easily through their lives and also to gain confidence when participating in these activities at school, which in turn could help with social maturity. As the Vineland scale in the second hypothesis confirmed, the locomotion skills and self-help dressing factor had a significant connection with BMI, thus it is safe to say that BMI and gross motor development have a direct effect on social maturity. Meanwhile, the fact that normal-weight adolescents had better results in gross motor skills was in agreement with Lopez et al. [11]. Furthermore, the findings of the first hypothesis also prove that overweight and obese children performed worse in performing fundamental movement skills, namely gross motor (12).

The results of the second hypothesis in self-help dressing is consistent with the study of Letsari et al. [16] that social immaturity in the group of obese children was more than normal-weight children. Although, it should be noted that the participants in the test were male children. However, the findings in the mentioned study are not consistent with the locomotion skills factor of our results, whereas overweight and normal-weight adolescents did not have a significant difference in their performance.

In addition, another inconsistency was with Verdejo-García, et al. [18], which showed that being overweight reduces social decision-making, and the connection between social communication and social maturity is reduced. Our findings in the second hypothesis did not show significant differences between the three groups (overweight, normal, and underweight) in the communication skills factor. The only factor that showed any connection between obesity and reduced social maturity was the self-help dressing factor, where overweight adolescents had a lower result compared to the other groups. This is connected with the lower health related quality of life score in obese children compared to their peers [31]. Meanwhile, underweight adolescents performed worse than the other groups in the locomotion skills factor. The method used in the locomotor skills of the vineland test was a movement too far from places on their own.

At last, our third hypothesis could not find significant differences among the group of overweight, normal-weight, and underweight adolescent girls in social maturity, and this finding is inconsistent with Madrona et al. [20]. The mentioned study confirmed that normal-weight girls performed significantly better than overweight girls in gross motor skills and balance, which is consistent with our findings regarding the gross motor skills in the first hypothesis. It also had a significant difference in social-emotional aspects (only in girls), where girls with normal weight had better social relationships, and girls with obesity acquired poorer results in social relationships.

Nonetheless, our study could not find any connection between the different BMI groups and emotional maturity. This result may be due to factors other than BMI, such as emotional instability in adolescence, smaller sample size, geographical and/or economic differences, not taking parental support, relations with friends or peers into account, etc. The main difference between the two studies is the age factor, which may have the biggest effect on the results; the mentioned study had participants between the age of 5 to 6 years, while our study consists of adolescent girls 12 to 14.5 years old. The greatest relative emotional instability is in the early adolescent years [32], thus this could have affected the results of our study. For further research, this test might provide another perspective on social maturity in correlation with BMI if it were performed on adults.

Our findings could provide noteworthy implications for middle and high school teachers and parents. Considering fundamental motor skills and their critical impact on adolescents’ daily lives, educators and parents should perceive BMI as an indicator of how gross motor skills can be developed, and take the reduced performance of overweight adolescent girls into account.

Despite the obtained results, however, this research had limitations that seem to be the basis for future research:

The statistical population was only limited to girls, it is better to conduct studies examining both genders.

To control the amount of physical activity in people using objective or subjective measurement methods.

The social and economic situation should be measured and controlled with comprehensive questionnaires and questions, and if necessary, it should be analyzed as an influencing factor.

To Investigate nutritional behaviors as an essential factor.

## Conclusion

According to the study results, it appears that gross motor skills of motor proficiency are affected by BMI, so, normal weight girls showed better performance on that. Therefore, it seems that by emphasizing normal weight in adolescent girls, improvement in gross motor skills can be expected. However, the research results showed that fine motor skills and motor coordination are not affected by body mass index. Therefore, future research is needed in order to reach the influencing factors of these variables. Also, normal-weight girls showed better performance on the self-help dressing and locomotion skills of social maturity in addition to this, under-wight girls in the self-help dressing and over-wight girls in the locomotion skills were superior to each other. In these two cases, it was also shown that the body mass index and normal weight can be clearly influential in the indicators of social maturity. Finally, emotional maturity did not show that it is influenced by body mass index, so future research can be a way to identify the influencing variables.

## Data Availability

The data of this study are available on request from the first corresponding author [S.H.Z.S].
